# Use of Length Heterogeneity Polymerase Chain Reaction (LH-PCR) as Non-Invasive Approach for Dietary Analysis of Svalbard Reindeer, *Rangifer tarandus platyrhynchus*

**DOI:** 10.1371/journal.pone.0091552

**Published:** 2014-03-11

**Authors:** Sungbae Joo, Donguk Han, Eun Ju Lee, Sangkyu Park

**Affiliations:** 1 Department of Biological Sciences, Ajou University, Suwon, South Korea; 2 School of Biological Sciences, Seoul National University, Seoul, South Korea; University of Central Florida, United States of America

## Abstract

To efficiently investigate the forage preference of Svalbard reindeer (*Rangifer tarandus platyrhynchus*), we applied length-heterogeneity polymerase chain reaction (LH-PCR) based on length differences of internal transcribed spacer (ITS) regions of ribosomal RNA (rRNA) to fecal samples from *R. tarandus platyrhynchus*. A length-heterogeneity (LH) database was constructed using both collected potential food sources of Svalbard reindeer and fecal samples, followed by PCR, cloning and sequencing. In total, eighteen fecal samples were collected between 2011 and 2012 from 2 geographic regions and 15 samples were successfully amplified by PCR. The LH-PCR analysis detected abundant peaks, 18.6 peaks on an average per sample, ranging from 100 to 500 bp in size and showing distinct patterns associated with both regions and years of sample collection. Principal component analysis (PCA) resulted in clustering of 15 fecal samples into 3 groups by the year of collection and region with a statistically significant difference at 99.9% level. The first 2 principal components (PCs) explained 71.1% of the total variation among the samples. Through comparison with LH database and identification by cloning and sequencing, lichens (*Stereocaulon* sp. and *Ochrolechia* sp.) and plant species (*Salix polaris* and *Saxifraga oppositifolia*) were detected as the food sources that contributed most to the Svalbard reindeer diet. Our results suggest that the use of LH-PCR analysis would be a non-invasive and efficient monitoring tool for characterizing the foraging strategy of Svalbard reindeer. Additionally, combining sequence information would increase its resolving power in identification of foraged diet components.

## Introduction

Svalbard reindeer (*Rangifer tarandus platyrhynchus*) lives on the high-arctic archipelago of Svalbard (74–80°N lat.) where snow and ice cover most of the local vegetation for 8 months of the year [1]–[3]. Because of the long winter period resulting in relatively lower forage availability and poor food quality, the reindeer have to replenish fat reserves for winter survival and fetus development during the summer period [4]–[7]. In addition, extreme seasonal variations in the high-arctic region impose strong pressures on arctic herbivores to feed on vegetation in a highly efficient manner to satisfy their energy requirements [7]. Previous studies have reported that reindeer are highly selective feeders and prefer lichens, mosses, graminoids, and various other plant species as food sources in the summer period [8], [9]. These preferences might be associated with their special nutritional needs (food quality) or plant biomass represented as food quantity [7], [10].

To investigate forage preference of Svalbard reindeer, previous studies have used either directly observed feeding behaviors by tracking the reindeer or analyzed diet composition from undigested contents in feces or rumen sampled in killed reindeer [4], [7], [10]. In general, these approaches have the advantage of providing direct information about diet and differentiating forage preferences by age and sex. In addition, directly assessing food consumption irrespective of food digestibility could be an obvious advantage of this approach. However, the direct-observation approach is very laborious because it allows monitoring of only a limited number of individuals at one time. Likewise, analysis of rumen contents requires direct handling of reindeers after killing and identification of undigested remains, which are difficult, laborious, and time consuming to perform.

Recently, molecular approaches have been widely used as non-invasive methods to study animal diet from feces or food remnants [11]–[15]. However, most of these approaches required laborious and time-consuming conventional sequencing methods, including cloning of PCR products and individual sequencing of clones [16]. The recent development of next-generation sequencing (NGS) methods has enabled the increased use of NGS-based methods on fecal samples to analyze diets [16]–[20]. The use of NGS technology for dietary analysis can provide an unprecedented amount of sequence data at lower costs than conventional molecular methods [21]. However, in spite of the advantages, application of NGS technology for reindeer diets may be pricy because available and preferred food sources for reindeer are limited at a given time and each fecal sample would contain only several short-term dietary compositions [8]-[9].

Applications of length-heterogeneity analysis by PCR (LH-PCR) to study forage preference on the basis of fecal samples can solve various difficulties typically encountered in conventional sampling methods. LH-PCR is widely applied, for example, to study the microbial diversity in natural ecosystems, and LH-PCR has been demonstrated to be an easy, fast, reliable, and highly reproducible method [22]–[26]. LH-PCR is capable of discriminating amplicons originating from different organisms on the basis of natural variation in the lengths of its DNA target regions [26]. Each peak in LH profiles represents distinct genotypes contributing to diet composition; peak numbers correspond to minimum richness of diet genotypes; and peak heights indicate abundance of each genotype [27].

Our study aimed to evaluate the potential applicability of an LH-PCR approach for dietary analysis of *R. tarandus platyrhynchus*. First, we constructed an LH-length database of potential food sources, including various vascular plants, mosses, lichens, and mushrooms representing the local flora in Svalbard. Second, we conducted LH-PCR of reindeer fecal samples collected from different sites in 2011 and 2012. Third, we determined the forage preference of Svalbard reindeer by comparing the LH-PCR profiles of collected fecal samples with the profiles in the newly constructed LH-length database.

## Materials and Methods

### A. Study sites and sample collection

**Figure 1:**
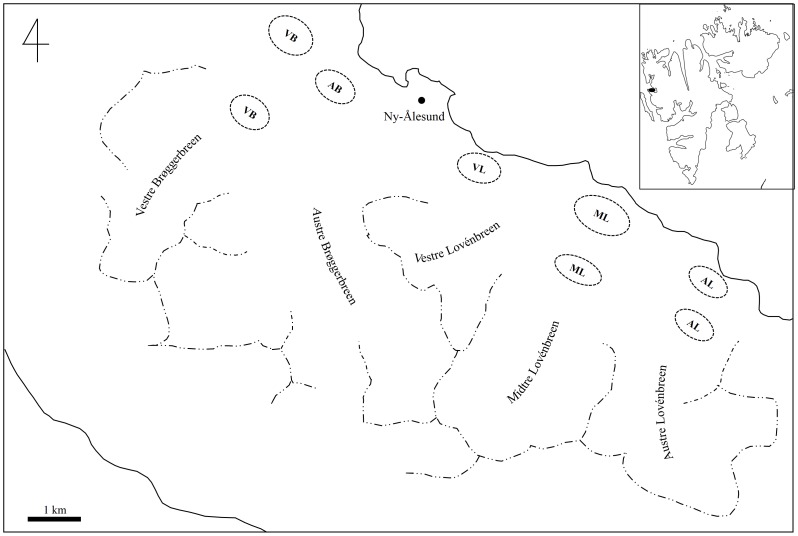
The study area, Ny-Ålesund (78°53'-78°55'N, 11°46'-12°11'E), northwest coast of Spitsbergen Island, Svalbard in Norway. Eight different sites were selected as sampling sites around two different glaciers. First letter of abbreviation represented directions: A: east (Austre), M: middle (Midtre), V: west (Vestre). Last letter of abbreviation represented two different glaciers: B: Brøggerbreen, L: Lovénbreen.

Our study was performed in Ny-Ålesund (78°53′–78°55′N, 11°46′–12°11′E), located at the northwest coast of Spitsbergen Island, Svalbard in Norway (Fig. 1), with a permission from the Governor of Svalbard and registered in Svalbard Science Forum (www.rcn.no/ssf; RIS ID: 4985). There are small research stations representing the most northerly human settlements, including Dasan Station of the Korea Polar Research Institute (KOPRI) in Ny-Ålesund. In this region, reindeer (*R. tarandus platyrhynchus*) are protected from hunting or any other human development such as tourism [1], [3]. The local vegetation includes short-growing plants, such as mosses, lichens (*Cetraria delisei*), the polar willow (*Salix polaris*), the purple saxifrage (*Saxifraga oppositifolia*), grasses and sedges, all of which contribute to the diet of Svalbard reindeer [2]. Eighteen fecal samples of *R. tarandus platyrhynchus* were collected during August in 2011 and 2012 from two different glacier areas: Brøggerbreen and Lovénbreen in Ny-Ålesund, where tourist visits are restricted (Fig. 1). All fecal samples were collected only on the ground from the approved area near the Dasan Station. In addition, various food sources such as common vascular plants and graminoids, mosses, and lichens were collected to construct an LH-length database and to determine forage preferences from reindeer feces. The fecal samples collected were transferred individually into polyethylene bags and kept at 4°C on ice during delivery to the laboratory. All samples were stored at -80°C until DNA extraction.

### B. Sample preparation and DNA extraction

DNA was extracted from feces using the QIAamp DNA Stool Mini Kit (Qiagen) following manufacturer’s protocols except for the lysis step. For sufficient homogenization, we added one or two 5-mm stainless steel beads (Qiagen) in the lysis step and mixed them by shaking on a Mixer Mill (Retsch, Germany) at 20 Hz for 1 min. Extracted DNA was eluted in 200 μl of AE buffer, and dilutions of 1∶10 were made in HPLC-grade H_2_O for use in subsequent PCRs. DNA extracts were stored at –20°C until further analyses.

### C. PCR amplification, cloning, and sequencing for construction of LH database

The universal ITS primer set comprising ITS3, 5′-GCATCGATGAAGAACGCAGC-3′ and ITS4, 5′-TCCTCCGCTTATTGATATGC-3′ was used for amplifying the ITS2 region of ribosomal RNA (rRNA) genes [28]. In each PCR amplification, 1 μl of extracted DNA was added to 24 μl of the amplification mixture, resulting in final concentrations of 1× Ex *Taq* Buffer, 1.5 mM of MgCl_2_, 0.2 mM of dNTPs, 0.2 μM of each primer, and 1 U of Ex *Taq* DNA polymerase (Takara, Japan), in a final reaction volume of 25 μl. PCR conditions were as follows: an initial denaturation at 95°C for 5 min, 45 cycles of denaturation at 95°C for 30 s; annealing at 50°C for 30 s; elongation at 72°C for 1min 30s, and a final extension step at 72°C for 7 min. PCR products amplified in the reaction were purified using Expin PCR SV Kit (GeneAll, Korea). Purified PCR products were ligated into the pGEM-T Easy Vector according to the manufacturer's protocols (Promega, USA) and transformed into DH5α chemically competent cells. Cells were plated in Luria–Bertani agar + ampicillin medium with 40 μl of X-gal solution (2% w/v) for antibiotic selection and blue-white screening. After the cloning step, 3 to 5 white colonies were selected and used in colony PCR for amplification with M13F and M13R primers. an initial denaturation at 95°C for 10 min, 35 cycles of denaturation at 94°C for 30 s; annealing at 55°C for 30 s; elongation at 72°C for 1 min and a final extension step at 72°C for 7 min. PCR products amplified in the reaction were purified using the Expin PCR SV Kit (GeneAll, Korea). Sequencing was conducted by a commercial sequencing service company (Macrogen, Korea). Each obtained DNA sequence was identified by BLASTN searches of the GenBank database. Sequence alignments and length calculations were conducted using the MEGA 5 program [29].

### D. LH analysis

For LH analysis, FAM-ITS3 and ITS4 were used in PCR amplifications. FAM-ITS3 was the ITS3 primer labeled on its 5′ end with the phosphoramidite fluorochrome 5-carboxyfluorescein (FAM). The buffer and reagent composition of each PCR reaction was the same as described above. For PCR cycling, conditions were modified to minimize amplification bias as follows: an initial denaturation at 95°C for 7 min, 30 cycles of denaturation at 95°C for 30 s; annealing at 50°C for 30 s; elongation at 72°C for 1min 30s, and a final extension step at 72°C for 7 min. Three PCR products amplified under identical conditions were combined and purified as described above. The LH analyses were conducted by a commercial company (Solgent, Korea) with an internal size standard (Genescan 500 ROX, Applied Biosystems) ranging from 35 to 500 bp, which covered most of the major DNA peaks. LH profiles were analyzed using the DAx software (Van Mierlo Software Consultancy, Netherlands).

### E. Statistical analysis

LH profiles of collected fecal samples were compiled and aligned to produce a large data matrix (15 observations × 221 peak variables). LH profile data were centered and standardized to relative abundance before conducting principal component analysis (PCA). We assigned “0” when a matching peak was absent. PCA was applied to the weighted covariance data matrix to reduce its dimensionality. Multivariate analysis of variance (MANOVA) was conducted on three groups (Lovénbreen, Ny-Ålesund in 2011 vs. Brøggerbreen, Ny-Ålesund in 2012 vs. Lovénbreen, Ny-Ålesund in 2012) according to between sampling years and sites using un-rotated PC scores. Analysis of variance (ANOVA) was used to test for statistically significant differences of major foraged components represented as relative peak area (expressed as %) on first 2 PC-axis, and *post hoc* tests were performed by using Tukey’s method (*P*<0.05). All statistical analyses were performed with S-Plus 8 for Windows (Insightful Corp., USA).

## Results

### A. Construction of LH database from reference samples and fecal samples

LH lengths were measured from 64 reference samples containing lichens, mushrooms, and plants and combined with BLASTN results (Table 1 and 2). LH lengths ranged from 333.4 bp for a lichen species (*Ochrolechia* sp.) to 494.1 bp for moss (*Polytrichum* sp.) (Table 1 and 2). Most of the samples identified as lichen had LH lengths of <350 bp. Mushrooms had LH lengths ranging from 369.3 bp for *Cortinarius saturninus* to 450.3 bp for *Russula silvicola*, and were divided into three groups by size difference. Plants and mosses had longer LH lengths, ranging from 364.7 to 494.1 bp, than the other predicted food species. In addition, 22 unique sequences were detected from collected fecal samples by selection after cloning (Table 1). Additionally, we conducted cloning with Sanger sequencing from collected 15 feces to identify foraged food sources and included in LH database (Table 2). Twenty-seven different sequences were detected including lichens (*C. delisei* and *Stereocaulon sp.*), mushrooms (*Hebeloma sp.* and *Cortinarius favrei*), and common vascular plants (*Salix polaris* and *Bistorta vivipara*). Several fungi (*Cladosporium* sp., *Friedmanniomyces endolithicus*, and *Thelebolus microsporus*) that do not form mushrooms or lichens were also detected from feces and they displayed LH lengths ranging from 329.8 to 406.3 bp.

**Table 1:** Constructed LH database for 32 vascular plants and 2 bryophyte species living in Ny-Ålesund (78°53'-78°55'N, 11°46'-12°11'E), Spitsbergen Island, Svalbard in Norway.

Sample ID	Species	Amplification length (bp)	Accession	Remarks
Sval_R1	*Koenigia islandica*	364.7	KC691698	
Sval_R2	*Braya glabella* ssp. *purpurascens*	367.5	KC691699	
Sval_R3	*Cochlearia groenlandica*	369.9	KC691700	
Sval_R4	*Draba alpina*	370.3	KC691701	
Sval_R5	*Carex nardina* ssp. *hepburnii*	370.6	KC691702	
Sval_R6	*Oxyria digyna*	371.8	KC691703	
Sval_R7	*Cardamine pratensis* ssp. *angustifolia*	372.7	KC691704	
Sval_R8	*Minuartia biflora*	384.5	KC691705	
Sval_R9	*Ranunculus pygmaeus*	387.2	KC691706	
Sval_R10	*Ranunculus hyperboreus* ssp. *arnellii*	388.5	KC691707	
Sval_R11	*Poa alpina var. vivipara*	390.9	KC691708	
Sval_R12	*Festuca* sp.	391	KC691709	
Sval_R13	*Deschampsia alpina*	391.1	KC691710	
Sval_R14	*Salix polaris*	391.2	KC691711	
Sval_R15	*Dryas octopetala*	394.3	KC691712	
Sval_R16	*Puccinellia vahliana*	395.2	KC691713	
Sval_R17	*Cerastium arcticum*	395.7	KC691714	
Sval_R18	*Trisetum spicatum* ssp. *spicatum*	396	KC691715	
Sval_R19	*Stellaria* sp.	396.4	KC691716	
Sval_R20	*Sagina nivalis*	397.6	KC691717	
Sval_R21	*Pedicularis hirsuta*	404	KC691718	
Sval_R22	*Bistorta vivipara*	407.9	KC691719	
Sval_R23	*Cassiope tetragona* ssp. *tetragona*	408.4	KC691720	
Sval_R24	*Luzula confusa*	409.4	KC691721	
Sval_R25	*Micranthes hieracifolia*	412.7	KC691722	
Sval_R26	*Micranthes foliolosa*	413.9	KC691723	
Sval_R27	*Saxifraga rivularis* ssp. *rivularis*	415.3	KC691724	
Sval_R28	*Saxifraga cespitosa*	417.4	KC691725	
Sval_R29	*Saxifraga oppositifolia* ssp. *oppositifolia*	418.5	KC691726	
Sval_R30	*Saxifraga aizoides*	419.1	KC691727	
Sval_R31	*Huperzia arctica*	424	KC691728	
Sval_R32	*Sanionia uncinata*	430.5	KC691729	Bryophyte
Sval_R33	*Papaver dahlianum*	433.7	KC691730	
Sval_R34	*Polytrichum* sp.	494.1	KC691731	Bryophyte

All of sequences on ITS2 region of rRNA gene were registered in Genbank (Accession number: KC691698-KC691731). They were arranged by the LH length.

**Table 2:** Constructed LH database between 30 different sequences containing collected potential food sources of Svalbard reindeer (*R. tarandus platyrhynchus*) and 27 sequences detected from fecal samples by cloning.

Sample ID	BLAST results			Amplification length (bp)	Occurrence of identified taxon on Svalbard	Origin of sequence
	Description	Identities	Gaps			
**Food sources**						
Sval_1	*Ochrolechia tartarea* (Li)	286/298(96%)	1/298(0%)	333.4	*Ochrolechia* sp.	
Sval_2	*Biatora carneoalbida* (Li)	296/297(99%)	1/297(0%)	333.7	Yes	
Sval_3	*Cetrariella delisei* (Li)	296/297(99%)	1/297(0%)	333.8	Yes	Feces
Sval_4	*Cetrariella fastigiata* (Li)	292/298(98%)	2/298(0%)	333.9	*Cetrariella delisei*	
Sval_5	*Cetrariella fastigiata* (Li)	293/297(99%)	1/297(0%)	334	*Cetrariella delisei*	Feces
Sval_6	*Ochrolechia tartarea* (Li)	297/298(99%)	0/298(0%)	334.5	*Ochrolechia* sp.	
Sval_7	*Stereocaulon tomentosum* (Li)	293/299(98%)	0/299(0%)	335.5	*Stereocaulon* sp.	Feces
Sval_8	*Umbilicaria decussata* (Li)	298/303(98%)	1/303(0%)	340.1	Yes	
Sval_9	*Umbilicaria umbilicarioides* (Li)	296/303(98%)	0/303(0%)	340.9	*Umbilicaria* sp.	
Sval_10	*Cladonia arbuscula* ssp. *beringiana* (Li)	194/194(100%)	0/194(0%)	345.4	*Cladonia arbuscula*	
Sval_11	*Cladonia borealis* (Li)	258/258(100%)	0/258(0%)	346.9	Yes	
Sval_12	*Cladonia grayi* (Li)	311/313(99%)	0/313(0%)	349.9	*Cladonia* sp.	
Sval_13	*Cortinarius saturninus* (Mu)	329/329(100%)	0/329(0%)	369.3	Yes	
Sval_14	*Cortinarius* sp. (Mu)	329/332(99%)	1/332(0%)	371.5	Potentially yes	
Sval_15	*Hebeloma testaceum* (Mu)	362/363(99%)	0/363(0%)	371.8	*Hebeloma* sp.	Feces
Sval_16	*Auricularia auricula-judae* (Mu)	285/351(81%)	17/351(4%)	382.2	Yes	
Sval_17	*Inocybe terrigena* (Mu)	305/357(85%)	17/357(4%)	385.4	Yes	
Sval_18	*Cortinarius favrei* (Mu)	349/351(99%)	0/351(0%)	390.5	Potentially yes	Feces
Sval_19	*Cortinarius trivialis* (Mu)	350/351(99%)	0/351(0%)	391	*Cortinarius* sp.	
Sval_20	*Salix bebbiana* (An)	359/360(99%)	0/360(0%)	391.1	*S. polaris*	Feces
Sval_21	*Cortinarius favre*i (Mu)	350/351(99%)	0/351(0%)	391.4	Yes	
Sval_22	*Cortinarius favre*i (Mu)	350/351(99%)	0/351(0%)	391.6	Yes	
Sval_23	*Cortinarius favre*i (Mu)	350/351(99%)	0/351(0%)	391.6	Yes	
Sval_24	*Entoloma aff. sinuatum* (Mu)	581/583(99%)	1/583(0%)	391.7	Yes	
Sval_25	*Cortinarius favre*i (Mu)	350/351(99%)	0/351(0%)	391.9	Yes	
Sval_26	*Salix herbacea* (An)	358/360(99%)	0/360(0%)	392.3	Yes	Feces
Sval_27	*Salix bebbiana* (An)	360/360(100%)	0/360(0%)	392.6	*S. polaris*	Feces
Sval_28	*Salix bebbiana* (An)	359/359(100%)	0/359(0%)	392.6	*S. polaris*	Feces
Sval_29	*Silene paradoxa* (An)	365/367(99%)	1/367(0%)	400.5	*Silene* sp.	Feces
Sval_30	*Inocybe leucoloma* (Mu)	360/361(99%)	0/361(0%)	401	Yes	
Sval_31	*Omphalina chionophila* (Mu)	362/363(99%)	0/363(0%)	403	Yes	
Sval_32	Sebacinales (Mu)	323/366(88%)	18/366(4%)	403	Potentially yes	
Sval_33	*Omphalina chionophila* (Mu)	362/363(99%)	0/363(0%)	403.2	Yes	
Sval_34	*Bistorta subscaposa* (An)	374/380(98%)	0/380(0%)	409.4	*Bistorta vivipara*	Feces
Sval_35	*Bistorta subscaposa* (An)	372/380(98%)	0/380(0%)	410.2	*Bistorta vivipara*	Feces
Sval_36	*Saxifraga fortunei var. alpina* (An)	309/325(95%)	6/325(1%)	414.3	*Micranthes hieracifolia*	Feces
Sval_37	*Polytrichum juniperinum* (Mo)	363/367(99%)	1/367(0%)	416.2	Yes	Feces
Sval_38	*Polytrichum juniperinum* (Mo)	363/367(99%)	1/367(0%)	416.8	Yes	Feces
Sval_39	*Saxifraga oppositifolia* (An)	345/346(99%)	0/346(0%)	419	Yes	Feces
Sval_40	*Saxifraga oppositifolia* (An)	345/345(100%)	0/345(0%)	419.5	Yes	Feces
Sval_41	*Saxifraga oppositifolia* (An)	343/345(99%)	0/345(0%)	419.6	Yes	Feces
Sval_42	*Saxifraga oppositifolia* (An)	345/347(99%)	1/347(0%)	419.8	Yes	Feces
Sval_43	*Saxifraga oppositifolia* (An)	345/345(100%)	0/345(0%)	420.9	Yes	Feces
Sval_44	*Saxifraga oppositifolia* (An)	345/346(99%)	0/346(0%)	420.9	Yes	Feces
Sval_45	*Saxifraga oppositifolia* (An)	345/346(99%)	0/346(0%)	421.3	Yes	Feces
Sval_46	*Lactarius luculentus var. laetus* (Mu)	404/417(97%)	9/417(2%)	448.7	Yes	
Sval_47	*Lactarius luculentus var. laetus* (Mu)	406/417(97%)	9/417(2%)	448.8	Yes	
Sval_48	*Russula laccata* (Mu)	409/409(100%)	0/409(0%)	449.1	Yes	
Sval_49	*Russula laccata* (Mu)	407/409(99%)	0/409(0%)	450.3	Yes	
**Non-food sources**						
Sval_50	Ericoid mycorrhizal sp. (Fu)	249/285(87%)	4/285(1%)	329.8		
Sval_51	*Cladosporium* sp. (Fu)	296/296(100%)	0/296(0%)	333.5		Feces
Sval_52	*Thelebolus microsporus* (Fu)	295/295(100%)	0/295(0%)	334.6		Feces
Sval_53	*Sporormiella vexans* (Fu)	257/257(100%)	0/257(0%)	336.6		
Sval_54	*Friedmanniomyces endolithicus* (Fu)	268/306(88%)	13/306(4%)	339.8		Feces
Sval_55	*Friedmanniomyces endolithicus* (Fu)	268/307(87%)	11/307(3%)	340.2		Feces
Sval_56	*Friedmanniomyces endolithicus* (Fu)	268/307(87%)	11/307(3%)	340.8		Feces
Sval_57	*Tomentella bryophila* (Fu)	356/368(97%)	0/368(0%)	406.3		

They were arranged by LH length. “Yes” indicates species found in Svalbard. Sequences obtained from feces by cloning represented as “Feces”. Each abbreviation represented as follows: An: angiosperms; Fu: other fungi, which do not form food sources like mushrooms and lichens; Li: lichens; Mo: mosses; Mu: mushroom-forming fungi.

### B. LH analysis

**Figure 2:**
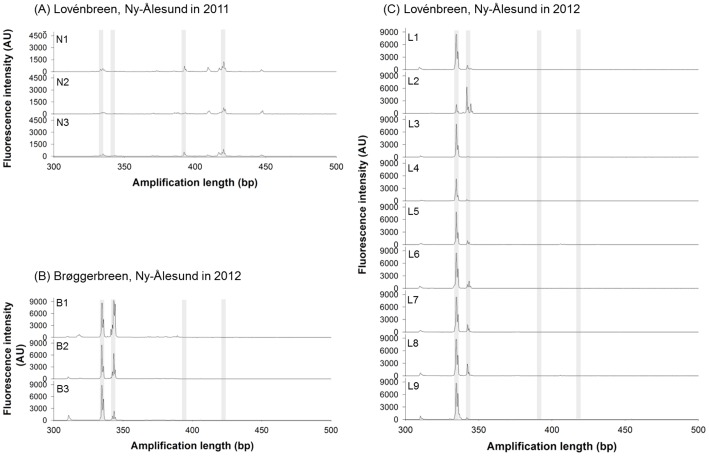
LH profiles of 15 fecal samples collected from different regions in between 2011 and 2012. (a) Lovénbreen, Ny-Ålesund in 2011; (b) Brøggerbreen, Ny-Ålesund in 2012; (c) Lovénbreen, N-Ålesund in 2012. Shaded bars on profiles represented major peaks, which were the most contributing variables on first two principal components in PCA analysis.

Among the 18 fecal samples collected, 15 resulted in successful PCR amplification, and they were used for LH analysis. Most LH profiles consisted of an abundant number of peaks, with an average profile having 18.6 peaks ranging in size from 100 to 500 bp (Fig. 2, Table 3). Most of the informative peaks were detected in the range of 300 to 500 bp in all LH profiles. The LH profiles from fecal samples collected in 2011 had higher number of peaks, 39 on an average, than the LH profiles from fecal samples collected in 2012. However, most peaks from the 2011 samples showed lower fluorescence intensity than the peaks in the 2012 samples. LH peaks of more than 400 bp in length were detected in all LH profiles from the 2011 samples. Fecal samples collected in 2012 showed LH profile patterns that were different from the LH profile patterns of the samples collected in 2011 (Fig. 2). Most of the LH profiles had the largest peaks at approximately 335 bp, and they were composed of two major peaks that were shorter than 350 bp.

**Table 3:** Number of LH peaks in LH profiles and estimated of diet composition on different sampling regions in between 2011 and 2012 (Lovénbreen, Ny-Ålesund in 2011 vs. Brøggerbreen, Ny-Ålesund in 2012 vs. Lovénbreen, Ny-Ålesund in 2012).

Sampling site	Number of samples	Number of LH peaks in profiles			Diet composition (relative peak area, %)				
		average	min	max	Lichens	Mushrooms	Angiosperms	Other fungi	Unclassified
Lovénbreen, Ny-Ålesund in 2011	3	36.7±4.5	32	42	7.4±1.9	1.6±0.8	59.8±8.0	5.2±3.5	2.3±1.5
Brøggerbreen, Ny-Ålesund in 2012	3	24.3±17.2	9	43	20.8±7.0	0.5±0.8	-	36.0±13.6	20.0±10.0
Lovénbreen, Ny-Ålesund in 2012	9	10.7±4.2	4	19	26.0±9.2	-	-	56.7±17.8	8.0±13.8

### C. Principal component analysis of LH profiles

**Figure 3:**
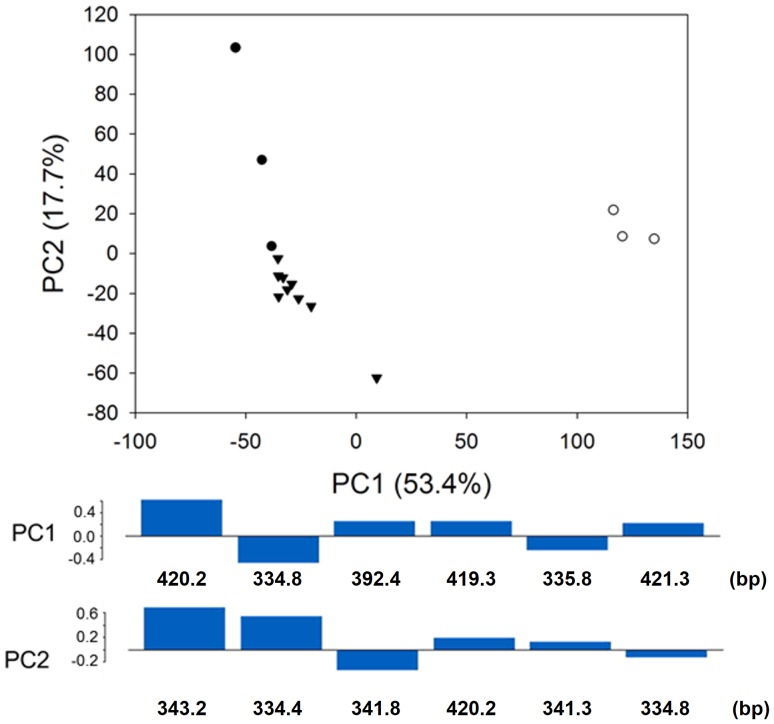
PCA results extracted from LH profiles of 15 fecal samples from collected from different regions in between 2011 and 2012. Bars represented loading values of the first principal component (PC1) and the second principal component (PC2). Open circles: Lovénbreen, Ny-Ålesund in 2011; Closed circles: Brøggerbreen, Ny-Ålesund in 2012; Closed inverted triangles: Lovénbreen, Ny-Ålesund in 2012.

PCA of LH profiles was performed to compare dietary components and resulted in the first two principal components (PCs) explaining 71.1% of the total variation among the profiles (PC1 for 53.4% and PC2 for 17.7%; Fig. 3). MANOVA on the PCA scores from the LH-profile comparisons showed that the PCA scores of 3 groups (Lovénbreen, Ny-Ålesund in 2011 vs. Brøggerbreen, Ny-Ålesund in 2012 vs. Lovénbreen, Ny-Ålesund in 2012) were statistically significantly different from each other (*P*<0.001). In particular, the PCA score plots showed that fecal samples obtained from different years were separated mainly by the first PC axis. Five of the six peaks that, according to their loading values, represented variables that contribute most to the first principal component, were assigned as lichens (*Stereocaulon* sp. and *Ochrolechia* sp.) and vascular plant species (*Salix polaris* and *Saxifraga oppositifolia*) on the basis of comparisons with the constructed LH database (Fig. 2 and 3, Table 1 and 2). Plant species were detected only in samples collected in 2011 (59.8% of total relative peak area, Table 3). One-way ANOVA results indicated that the percentage of relative areas for peaks corresponding to lichen species was statistically significantly different among 3 different groups separated on the first PC axis, according to the sampling year and region *(P* = 0.008).

## Discussion

Detailed and accurate information on forage preference in accordance with seasonal and regional characteristics are very important to understand energy requirement of Svalbard reindeer (*R. tarandus platyrhynchus*) as related to food quality and food quantity [4], [7], [30]–[33]. Our LH-PCR approach for fecal dietary analysis from feces could detect several preferred food species through comparison with a constructed LH database and sequencing results. This strategy would increase resolving power in identification and efficiently detect various food sources from collected fecal samples.

Svalbard reindeer are known as selective feeders, preferring lichens over grasses, and preferring grasses over mosses [9]. Our results of the LH-PCR supported the results of the previous study that lichen species may be one of important and preferable food sources in the study sites in Svalbard, especially during the summer season. More than 20% of the total peak area represented lichen species in 2012. In the 2011 samples, lichen species also represented 7.4% of total diet composition although the peaks identified as plant species contributed the highest percentage of the total peak area (Table 3). In addition, our results showed that LH-PCR on fecal samples could detect both seasonal and regional differences of feeding preferences. Although we collected reindeer feces from only three different regions and only in the summer season, significant differences in LH peak numbers and relative peak areas (%) varying by sampling year and site suggested an abundance of different food sources foraged by reindeer (Table 2 and 3). These results indicate that the observed LH profiles reflect different reindeer forage preferences that are affected by the composition of the vegetation available in the different regions and annual season.

Terrestrial mammals may make use of intertidal zones as forage sites during resource-restricted periods [34]. Hansen and Aanes [35] reported that kelp or seaweed were foraged by Svalbard reindeer (*R. tarandus platyrhynchus*) under extreme weather conditions such as icing associated with heavy rain-on-snow events. Most of these studies relied on direct observations of feeding behavior [34], [35]. We suggest that LH-PCR analysis of fecal samples can be useful for studying foraging strategy in the winter season. In fact, we obtained 112 sequences deposited in GenBank from 4 kelp species and 7 seaweed species growing in the Svalbard or polar regions [36]–[39]. We then predicted amplification lengths from these sequences on the basis of a calculated relationship between predicted and measured amplification lengths by using the information of the LH database constructed in this study (*y* = 0.96*x* + 8.95, R^2^ = 0.98). This *in silico* analysis indicated that LH amplification from kelp and seaweed should result in DNA fragments with lengths in the range of 182.7 to 364.1 bp, which is an appropriate range for LH-PCR analysis.

We considered that the ITS gene may be appropriate marker for dietary analysis of reindeer. Reindeer preferred lichens, mushrooms, and mosses as well as various other plant species as food sources. To detect all potential food sources from feces using LH-PCR, target genes must have enough resolution for various potential food sources and enough length variations. The ITS gene of nuclear ribosomal DNA is commonly sequenced in fungi than any other region of DNA and recommended as representatives of fungal DNA barcode [40], [41]. Additionally, Chen and his colleague tested that ITS2 region represents the most suitable region for DNA barcoding applications compared to seven candidate DNA barcode [42]. They tested the discrimination ability of ITS2 in more than 6600 plant samples and identification success rate was recorded 92.7% at the species level although this study was conducted on medicinal plants [42]. Also, It is well known that the ITS gene shows highly length variation compared to other conservative regions [43].

To better interpret forage preferences from fecal samples by using LH profiles, several limitations should be considered. First, it is necessary to construct an additional LH database for food sources used by reindeer not included in this study. Especially the construction of a local LH database would be useful to increase the resolution of the LH-PCR analysis. However, despite these efforts, it remains difficult to identify a food source reliably if the difference in LH length is small between foraged species (Table 1). Therefore, we recommend to apply the LH-PCR analysis in parallel with vegetation surveys on the study area to facilitate a more complete dietary analysis. Second, for technical reasons, the number of PCR cycles or template DNA concentration could affect the results of the LH-profile analysis because of kinetic bias during PCR amplification. In addition, lower DNA concentration in template can reduce detection rate of foraged diet components [44]. However, it has been proposed that decrease of PCR cycles could reduce kinetic bias in the PCR and give highly reproducible data [23], [26]. Based on our preliminary experiments, we decided 30 cycles as an appropriate number of cycles for analyzing the fecal samples. Additionally, in this study, we could not compare the change of LH-PCR pattern according to the template DNA concentration because DNA concentration extracted from feces showed lower than other organisms, in general. In further study, obtaining high template DNA would help to increase detection rate and reproducibility. Third, collection of fresh or recently excreted fecal samples may be helpful to increase prey DNA detection success. Decrease in prey detection success were observed when feces were exposed to rain and ultra violet (UV) radiation [12], [45]. DNA from feces exposed for long time to environmental conditions were more degraded by various reasons, such as environmental factors (rain and ultra violet (UV) radiation), enzymatic activities, consumed by microbe and so on [12], [46]. In this study, 15 feces were successfully amplified among the 18 fecal samples collected. When we collected fecal samples, all fecal samples were collected without selection by time after excretion. Consideration of time after defecation will increase successful recover rate of prey DNA from feces. Finally, combination with specific blocking primers will selectively prevent amplification of DNA from fungal species that are not reindeer food sources in further study. [47]–[50]. Such an application of blocking primers will increase the resolution and the accuracy of analyses for discriminating preferred food sources.

Most previous studies involving dietary analysis require direct handling of animals and result in difficulties in the identification of food sources from remains. Our results show that application of LH-PCR analysis would complement the methodological limitations of the traditional approaches. Although there are several limitations to this approach as mentioned above, LH-PCR could be complemented by expanding an LH database for potential food sources and increasing resolving power in identification. We believe that the use of LH-PCR analysis would be an ethical and efficient monitoring tool for investigating the foraging strategies of Svalbard reindeer.
